# Micro-geographic variation in antigenic diversity of PfEBA-175 region II in asymptomatic *Plasmodium falciparum* infections in Tanzania

**DOI:** 10.3389/fimmu.2025.1656267

**Published:** 2025-10-24

**Authors:** Jadidan Hada Syahada, Wang-Jong Lee, Hojong Jun, Johnsy Mary Louis, Fadhila Fitriana, Fauzi Muh, Feng Lu, Md Atique Ahmed, Sunghun Na, Wanjoo Chun, Won Sun Park, Bo-Young Jeon, Eun-Teak Han, Jim Todd, Alphaxard Manjurano, Winifrida Kidima, Ernest Mazigo, Se Jin Lee, Jin-Hee Han

**Affiliations:** ^1^ Department of Medical Environmental Biology and Tropical Medicine, School of Medicine, Kangwon National University, Chuncheon, Republic of Korea; ^2^ Department of Epidemiology and Tropical Diseases, Faculty of Public Health, Universitas Diponegoro, Semarang, Indonesia; ^3^ Department of Pathogenic Biology and Immunology, School of Basic Medical Sciences, Faculty of Medicine, Yangzhou University, Yangzhou, China; ^4^ Malaria Division, Indian Council of Medical Research (ICMR)-Regional Medical Research Centre, Dibrugarh, Assam, India; ^5^ Department of Obstetrics and Gynecology, Kangwon National University Hospital, Chuncheon, Republic of Korea; ^6^ Department of Pharmacology, School of Medicine, Kangwon National University, Chuncheon, Republic of Korea; ^7^ Department of Physiology, School of Medicine, Kangwon National University, Chuncheon, Republic of Korea; ^8^ Department of Biomedical Laboratory Science, College of Software and Digital Healthcare Convergence, Yonsei University, Wonju, Republic of Korea; ^9^ Department of Population Health, London School of Hygiene and Tropical Medicine, London, United Kingdom; ^10^ Department of Biostatistics, Catholic University of Health and Allied Sciences (CUHAS), Mwanza, Tanzania; ^11^ Department of Parasitic Diseases, National Institute for Medical Research, Dar es Salaam, Tanzania; ^12^ Department of Zoology, College of Natural and Applied Sciences, University of Dar es Salaam, Dar es Salaam, Tanzania; ^13^ Institute of Medical Sciences, Kangwon National University, Chuncheon, Republic of Korea

**Keywords:** *Plasmodium falciparum*, PfEBA-175, blood-stage malaria vaccine, genetic diversity, antigenicity

## Abstract

**Background:**

Malaria remains a major public health burden, and the development of effective blood-stage vaccines is complicated by extensive antigenic variation in *Plasmodium falciparum* antigens. PfEBA-175, a leading vaccine candidate, mediates erythrocyte invasion by binding to glycophorin A via its region II (RII), which is known to be highly polymorphic.

**Methods:**

We examined the genetic diversity and antigenicity of PfEBA-175 region II (RII) in 172 asymptomatic *P. falciparum* isolates collected from Geita and Kigoma, Tanzania. Sequence diversity was assessed through nucleotide diversity (π) and analysis of selection pressure using d_N_-d_S_ and Fu’s Fs tests. A recombinant PfEBA-175 RII protein was expressed to evaluate antibody response in plasma samples.

**Results:**

Sequence analysis revealed high nucleotide diversity (π = 0.00359 ± 0.00012), with evidence of adaptive evolution driven by immune pressure (d_N_-d_S_ = 3.15, *p* < 0.001), and sign of recent population expansion based on Fu’s Fs value (-30.614). A recombinant RII protein exhibited high antigenicity, with an average seropositivity of 84.8%, although rates varied across villages, ranging from 95.4% in Rwantaba to 50.0% in Bunyambo. The antibody response was positively correlated with age (*ρ* = 0.333, *p* < 0.001), but not with parasitemia or gender. Several important amino acids substitutions, including K478N, K481I, and L482V, were located within B-cell epitopes targeted by the invasion-inhibitory monoclonal antibody R217, and N577K, found at the dimer interface, had little effect on naturally acquired immune responses. However, several charged amino acid substitutions including D168H, T198K, K275I, K448N, and D619H influenced natural acquired antibody recognition.

**Conclusion:**

Despite substantial polymorphism, the glycan-binding residues of PfEBA-175 RII remain conserved, and its high antigenicity across diverse geographic and demographic contexts supports its continued evaluation as a blood-stage vaccine candidate. These findings highlight the importance of accounting for naturally occurring antigenic variation in malaria vaccine development.

## Introduction

Malaria remains a significant global health concern despite intensified control measures in recent years (1). It is caused by protozoan parasites of the genus *Plasmodium*, with *P. falciparum* being the deadliest of the five species that infect humans. According to the World Malaria Report 2024, there were an estimated 263 million malaria cases worldwide in 2023, an increase of 11 million compared to 2022, resulting in approximately 597,000 deaths (2). The WHO African Region accounted for the majority of global burden, contributing 94% of cases and 95% of deaths (2). Tanzania is among the countries most affected, responsible for 3.3% of global cases and 4.4% of malaria-related deaths in 2023 (2). The goal of global malaria control efforts has shifted from reducing malaria morbidity and mortality to the complete eradication of the disease (3, 4). Strategies such as insecticide-treated bed nets, indoor residual spraying, and effective antimalarial drugs have yielded some progress (5). However, the continued circulation of *P. falciparum* in endemic communities has accelerated the emergence and spread of drug resistance strains, undermining current treatment strategies. With the increasing threat of drug resistance, vaccines targeting the blood-stage are expected to play an increasingly critical role in integrated malaria control and elimination efforts.

The invasion of erythrocytes by merozoites is a key step in malaria pathogenesis and remains a major target for blood-stage vaccines (6). This process depends on coordinated interactions between parasite ligands and host receptors, and disrupting these interactions could prevent the parasite from entering host cells, thereby blocking the onset of clinical malaria (6). Blood-stage vaccine aims to inhibit merozoite invasion into erythrocytes, seeking to reduce morbidity, mortality, and transmission in malaria-endemic regions, while also alleviating the burden of severe and asymptomatic infections (7, 8). During the merozoite invasion, the parasite establishes strong interaction with specific receptors on host erythrocyte. Two major protein families, erythrocyte binding-like (EBL) and reticulocyte binding-like (RBL), have been identified as key ligands initiating these tight interactions and are considered promising vaccine target (9). Among the EBL family, *P. falciparum* erythrocyte binding antigen-175 (PfEBA-175) has emerged as a strong candidate for a blood-stage vaccine (10, 11). Antibodies against PfEBA-175 can block its interaction with the host receptor glycophorin-A (GpA), thereby preventing merozoite invasion (12). The *pfeba-175* gene is located on chromosome 7 and is composed of four exons. It is divided into seven regions (RI-RVII), with three conserved cysteine-rich domains found in Region II (F1 and F2) and Region VI (C-terminal) (13). PfEBA-175 RII, which features the erythrocyte binding-like domains within F1 and F2, plays a crucial role in GpA binding by promoting erythrocyte membrane deformability, a process for successful merozoite invasion (6, 14). Unlike other regions of PfEBA-175, region II directly mediates the functional interaction with host erythrocytes, making it a prime target for invasion-inhibitory antibodies. PfEBA-175 RII has been shown to elicit strong antibody responses and to be recognized by antibodies from individuals with naturally acquired immunity in endemic regions (15, 16). Furthermore, elevated antibody levels against PfEBA-175 RII have been correlated with protection against malaria, although the strength of this association appears to vary depending on transmission intensity (15, 16).

However, despite its promise as a vaccine candidate, PfEBA-175 RII exhibits substantial genetic diversity, primarily driven by immune selection pressure (17, 18). Such polymorphisms present a major challenge for vaccine development, as they may reduce vaccine efficacy by allowing the parasite to evade vaccine-induced immune responses (19). Recent studies have highlighted the importance of characterizing genetic polymorphisms in PfEBA-175 RII to inform rational vaccine design (20). An additional challenge is the high prevalence of asymptomatic infections, which act as silent reservoirs of transmission and often evade detection by conventional diagnostic methods (21). Persistent asymptomatic infections may further contribute to the maintenance and selection of diverse parasite variants, exerting sustained immune pressure on key antigens like PfEBA-175 RII over time (20). The persistence of asymptomatic infections may modulate host immune responses and complicate the assessment of vaccine efficacy. (22, 23). Additionally, assessing the antigenicity of circulating variants across different endemic settings in essential to ensure sustained vaccine effectiveness (24–26). Addressing asymptomatic malaria is thus crucial not only for transmission control but also for the successful development and deployment of blood-stage vaccines (27).

Given these challenges, the present study aimed to evaluate the feasibility of a PfEBA-175 RII-based vaccine by directly comparing genetic diversity and antigenicity across micro-geographical regions in malaria-asymptomatic individuals from high-endemic areas of Tanzania. While previous studies have investigated genetic diversity or antigenicity independently, few have examined both aspects of asymptomatic infections. Asymptomatic carriers, often overlooked in vaccine research, are critical to understanding transmission dynamics and the overall performance of malaria vaccines. By focusing on this population, this study provides important background into the potential of PfEBA-175 RII as viable vaccine target in regions with high endemicity.

## Methods

### Study site selection

**Figure 1:**
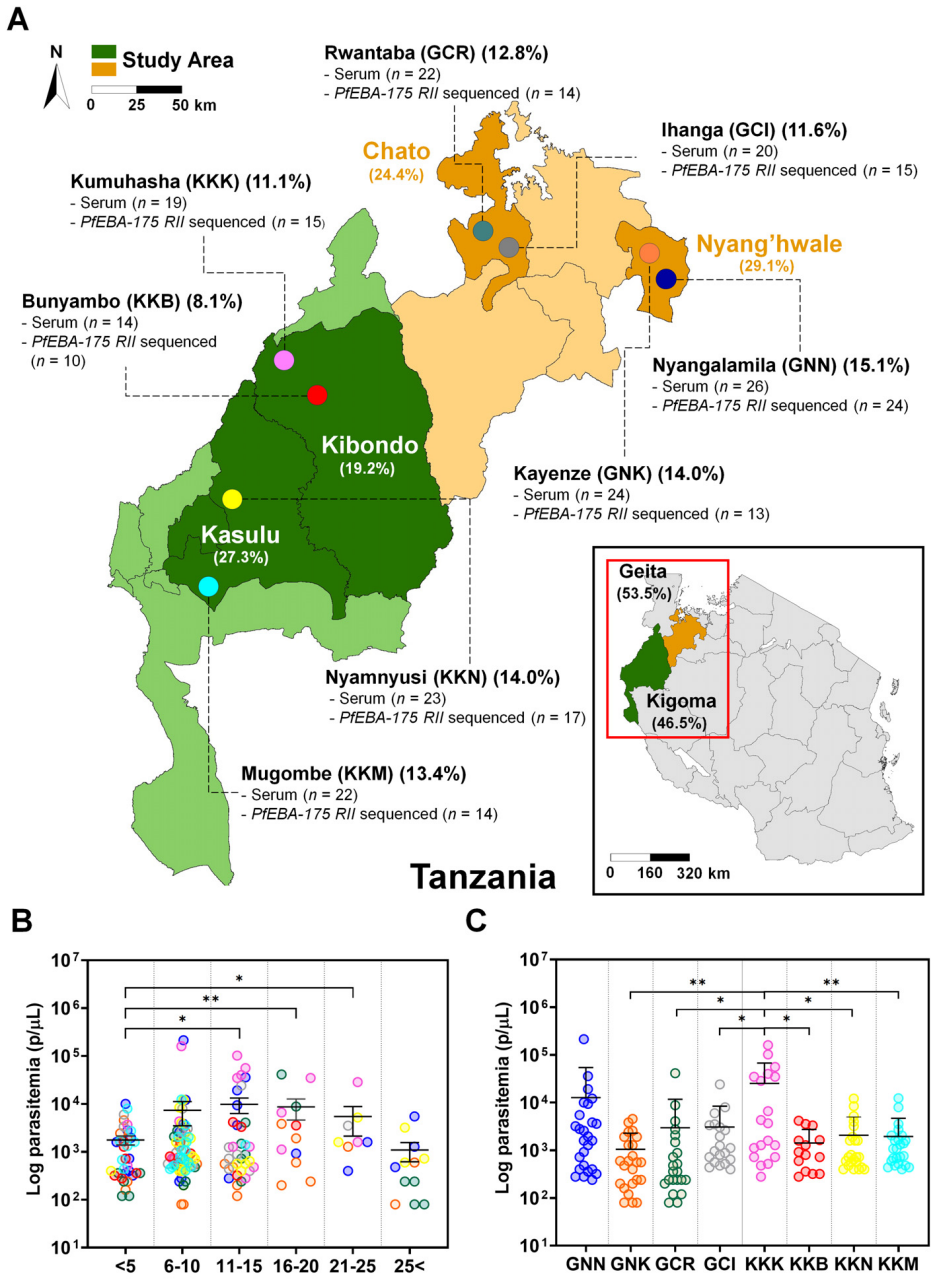
Study site overview and baseline characteristics of asymptomatic *Plasmodium falciparum* infections. **(A)** Map of Tanzania indicating the study sites in Geita and Kigoma regions, selected for their high *P. falciparum* prevalence in 2022. Two districts were selected from each region, with two villages per district. Geita contributed 53.5% of participants (Chato 24.4% [GCR 12.8%, GCI 11.6%]; Nyang’hwale 29.1% [GNK 14.0%, GNN 15.1%]). Kigoma contributed 46.5% (Kasulu 27.3% [KKM 13.4%, KKN 14.0%]; Kibondo 19.2% [KKB 8.1%, KKK 11.1%]). Percentages represent the proportion of total participants from each location. Samples were used for *pfeba-175 RII* sequencing (*n* = number successfully sequenced) and antigenicity analysis (serum *n* = number of samples tested). **(B)** Log-transformed parasitemia levels (parasites/µL) by age group. Parasitemia was significantly higher in the 11–15, 16–20, and 21–25-year age groups compared to children under 5 years (*p* < 0.05, unpaired Student’s t-test). No significant differences were observed between the under-5 group and individuals aged 6–10 or over 25 years. Data are shown as mean ± standard deviation (SD). Asterisks indicate statistical significance (*p < 0.05 and **p < 0.01). **(C)** Log-transformed parasitemia levels (parasites/µL) across the eight study villages. Kumuhasha (KKK) village showed a significantly higher mean parasitemia (Mean ± SD: 25,244 ± 42,248 parasites/µL) than other villages (GNK, GCR, GCI, KKB, KKN, KKM), as determined by unpaired Student’s *t*-test. Significant differences are denoted by asterisks (*p < 0.05) or double asterisks (**p < 0.01).

This study was conducted in two regions of Tanzania, Geita in the northwest and Kigoma in the west, where malaria prevalence was reported at 13% in both regions in 2022 (21). Based on high *Plasmodium falciparum* endemicity, two districts were randomly selected from each region, Nyang’hwale and Chato in Geita, and Kibondo and Kasulu in Kigoma. Subsequently, two villages were randomly selected from each district (**Figure 1A**). In total, eight villages from high-endemic micro-geographical areas were included in the study.

### Clinical sample isolates collection

Peripheral blood samples were collected between December 2022 and July 2023 from participants residing in the selected villages. The clinical sample collection procedures were described previously (21). Briefly, whole blood was obtained from asymptomatic individuals infected with *P. falciparum* during household visits conducted by the research team. After obtaining informed consent, demographic information was recorded using a standardized questionnaire. Individuals were classified as asymptomatic if they had no fever, no malaria-related symptoms in the previous 4–5 days, had not taken any antimalaria drugs within the past seven days, and had a positive microscopic diagnosis for parasitemia at any level.

Malaria infection was initially assessed using the Bioline™ Malaria Ag *P.f*/Pan rapid diagnostic test (RDT) (Abbott, Chicago, IL, USA). Thin blood smears were prepared by two trained microscopists, stained with Giemsa, and examined under a light microscope. Parasitemia was determined by counting parasites per 200 white blood cells, in accordance with the WHO Malaria Microscopy Standard Operating Procedures. As per national malaria treatment guidelines in Tanzania, asymptomatic individuals who tested positive for *P. falciparum* were treated with artemether-lumefantrine.

Following diagnosis, serum was separated and stored at -20 °C at a nearby health facility laboratory, while whole blood was preserved as dried blood spots (DBS) on Whatman 3MM filter paper. The serum and DBS samples were later transported to Kangwon National University for molecular diagnosis by qPCR, *pfeba-175 RII* gene sequencing, and antigenicity assessment.

A total of 172 whole blood samples were collected and processed for humoral immune response screening by protein microarray. However, only 122 (70.9%) samples yielded high-quality *pfeba-175 RII* sequences due to low parasitemia and suboptimal DBS quality. For negative controls in the protein microarray, healthy serum samples (*n* = 64) were obtained from children under 10 years of age residing in non-endemic regions of the Republic of Korea during routine health examinations at Kangwon National University Hospital.

### Genomic DNA extraction and *pfeba-175 RII* gene amplification

Genomic DNA was extracted from a single dried blood spot (50 μL) using the QIAamp DNA Mini Kit (QIAGEN, Hilden, Germany), following the manufacturer’s instructions, with a final elution volume of 50 μL. Extracted DNA was stored at -20 °C until use.

The *pfeba-175 RII* gene was successfully amplified and sequenced from 122 samples collected from high-endemic villages in Tanzania. PCR was performed to amplify the target region for downstream sequencing and analysis. Target-specific primers covering the entire coding region were designed based on the *pfeba-175 RII* gene (PF3D7_0731500) (**Supplementary Table S1**). Each 20 µL PCR reaction contained KOD One PCR Master Mix (Toyobo, Osaka, Japan), 1 µL of genomic DNA, 16 µL of distilled deionized water (DDW), and 1 µL of each 10 μM primer. The thermal cycling conditions were as follow, initial denaturation at 98°C for 7 minutes, 45 cycles of 98°C for 10 seconds (denaturation), 60°C for 5 seconds (annealing), and 68°C for 10 seconds (extension), followed by a final extension at 68°C for 10 minutes. Amplicons were visualized by electrophoresis on a 1.2% agarose gel and purified using a PureLink™ PCR Purification Kit (Invitrogen, Waltham, MA, USA). The purified amplicon was subjected to Sanger sequencing using the PCR forward primer and sequencing reverse primer on an ABI3730xl DNA analyzer (Nbit, Chuncheon, Republic of Korea) (**Supplementary Table S1**). Full-length *pfeba-175 RII* gene sequences were assembled using SnapGene v2.3.2 and the Lasergene 11 package, following chromatogram inspection for quality validation. The rare and novel genetic variants were confirmed by three independent sequencing experiments. The complete *pfeba-175 RII* sequences have been deposited in GenBank (accession numbers: PV683895 - PV684016).

### Genetic diversity and natural selection

The average number of nucleotide differences per site (*π*) was calculated to assess the genetic diversity of the *pfeba-175 RII* gene. Sequence diversity within the population was evaluated based on the number of polymorphic sites, number of haplotypes, and haplotype diversity (Hd) were computed using DnaSP v5.10 (28). To investigate potential signatures of natural selection, several neutrality tests were conducted, including Tajima’s D, Fu and Li’s D* and F*, and Fu’s Fs. Under the neutral theory of evolution, Tajima’s D is expected to be zero. Significantly negative values may indicate recent population expansion or purifying selection, while significantly positive values suggest balancing selection or a population bottleneck (29). Fu and Li’s D* and F* tests further assess deviations from neutrality, where significantly negative values typically indicate recent population growth with an excess of rare alleles, whereas significantly positive values suggest population contraction or the influence of background selection (30). Fu’s Fs test assesses demographic expansion by evaluating haplotype distributions. Negative Fs values indicate an excess of rare haplotypes, which may result from recent population expansion or genetic hitchhiking (31). To further explore selection pressure, the number of synonymous substitutions per synonymous site (d_S_) and nonsynonymous substitutions per nonsynonymous site (d_N_) were calculated using the Nei and Gojobori method with 1,000 bootstrap replicates in Mega 11 software. A d_N_-d_S_ greater than zero suggests positive selection, while less than zero suggests purifying selection. In addition, the McDonald-Kreitman (MK) test was performed to compare within-species polymorphisms with between-species divergence. For this analysis, *P. reichenowi* EBA-175 RII (PRCDC_0728300), a member of the *Laverania* clade closely related to *P. falciparum*, was used as an outgroup. DnaSP v5.10 was used to perform the MK test and generate relevant summary statistics, including the neutrality index (NI), which further supports inference of selection. Haplotype clustering patterns were visualized using the median-joining method in Network 10.1, while haplotype diversity was analyzed using DnaSP v5.10.

### Tertiary structure modelling and visualization

The tertiary structure of PfEBA-175 RII was retrieved from the Protein Data Bank (PDB ID: 1ZRO), which represents the PfEBA-175 RII crystallized in the presence of α2, 3-sialyllactose (6). Non-synonymous mutations were mapped and visualized onto the structure using Chimera X 1.9, with particular attention to those located at or near the interaction interface between PfEBA-175 RII and the host receptor.

### Recombinant protein expression

The recombinant PfEBA-175 RII (amino acids 152-743, PF3D7_0731500) was codon-optimized for mammalian expression, corresponding to a molecular weight of approximately 81.2 kDa. The gene was amplified using gene-specific cloning primers (**Supplementary Table S1**) and cloned into the pTT5 mammalian expression vector with a C-terminal 6x His tag using the In-Fusion^®^ HD Cloning Kit (Clontech, Mountain View, CA, USA). Expression was performed in HEK293-6E cells via transient transfection using linear polyethylenimine hydrochloride (PEI). After 5 days of culture at 37°C in a humidified incubator with 5% CO_2_, the secreted recombinant protein in the culture supernatant was harvested. The recombinant protein was purified using Ni-NTA agarose resin (QIAGEN) and eluted with buffer containing 300 mM imidazole, 50 mM HEPES, 5% glycerol, and 150 mM NaCl. Purity was assessed by SDS-PAGE (4-12% Bis-Tris gradient gel, Invitrogen) and Coomassie Brilliant Blue staining (Sigma-Aldrich, St. Louis, MO, USA).

### Protein microarray for antigenicity screening

Protein microarray analysis was performed to evaluate the antigenicity of PfEBA-175 RII. Three-aminopropyl coated glass slides were prepared as previously described (25). PfEBA-175 RII was printed onto each slide at an optimal concentration of 25 ng/µL per spot, followed by incubation for 2 hours at 37°C. After incubation, the slides were blocked for 1 hour at 37°C with 5% BSA in PBS-T (0.1% Tween-20). Sera from asymptomatic *P. falciparum*-infected individuals and healthy controls were diluted 1:25 in PBS-T and applied to each slide in duplicate for 1 hour at 37°C. IgG reactivity was visualized using 10 ng/µL of Alexa Fluor 546-conjugated goat anti-human IgG (Invitrogen) in PBS-T for 1 hour at 37 °C. Array slides were scanned using the InnoScan 300 scanner (Innopsys, Carbonne, France). The threshold for a positive response was defined as the mean fluorescence intensity (MFI) of healthy control plus two standard deviations.

### Epitope-specific reactivity profiling by amino acid substitution mapping

Mean fluorescence intensity (MFI) values associated with distinct amino acid polymorphisms at each residue were quantitatively aggregated and represented as a heatmap. The resulting data matrix was organized with amino acid substitutions as rows and the corresponding residue positions as columns. Visualization employed hierarchical structuring of antigenic variation patterns using the seaborn Python package with the rainbow colormap. Annotated mean MFI values within each cell enabled precise assessment of immune reactivity differences driven by specific amino acid changes.

### Population genetic statistical analysis

Group comparisons for protein microarray data were performed using unpaired Student’s *t*-tests. Associations between antigenicity and demographic variables (gender, age, parasitemia) were assessed by Spearman correlation (*ρ*). A *p*-value < 0.05 was considered statistically significant. Data were analyzed and visualized using SigmaPlot v12.0 (Systat Software Inc., San Jose, CA, USA) and GraphPad Prism v8 (GraphPad Software, San Diego, CA, USA) software.

### Ethics statement

The National Institute for Medical Research (NIMR), a branch of Tanzania’s Ministry of Health (MoH), provided ethical guidelines and approved methods for the collection of all clinical samples (NIMR/HQ/R.81/Vol.IX/4114). The Kangwon National University Institutional Review Board approved the secondary use of clinical samples for further processing and experimental protocols (KWNUIRB-2022-06-008). Informed consent was obtained in writing from all adult participants, whereas for child participants, consent was secured from their parent or legal guardian.

## Results

### Statistical profiling of the study population

This study involved collecting *Plasmodium falciparum* isolates from asymptomatic participants in the Geita and Kigoma regions of Tanzania, both of which were identified as high malaria burden in 2022 (21). Geita contributed 53.5%, comprising Chato 24.4% (GCR 12.8%; GCI 11.6%) and Nyang’hwale 29.1% (GNK 14.0%; GNN 15.1%), whereas Kigoma contributed for 46.5%, with contributions from Kasulu 27.3% (KKM 13.4%; KKN 14.0%) and Kibondo 19.2% (KKB 8.1%; KKK 11.1%) (**Figure 1A**).

Parasitemia levels and demographic factors were compared across asymptomatic individuals (**Supplementary Table S2**). No significant differences in parasitemia were observed between children under 5 years of age and those aged 6–10 or over 25 years. In contrast, parasitemia was significantly higher in the 11-15, 16-20, and 21-25-year age groups compared to children under 5 years (**Figure 1B**). Regional comparisons revealed significant variation in parasitemia levels. Kumuhasha (KKK) village exhibited a significantly higher mean parasitemia (Mean ± S.D., 25,244 ± 42,248 parasites/µL) than all other study villages except Nyangalamila (GNN) (**Figure 1C**). Although other regions showed lower mean parasitemia levels with considerable variability, no statistically significant differences were observed among them.

### Genetic diversity and structural mapping of PfEBA-175 RII

**Figure 2:**
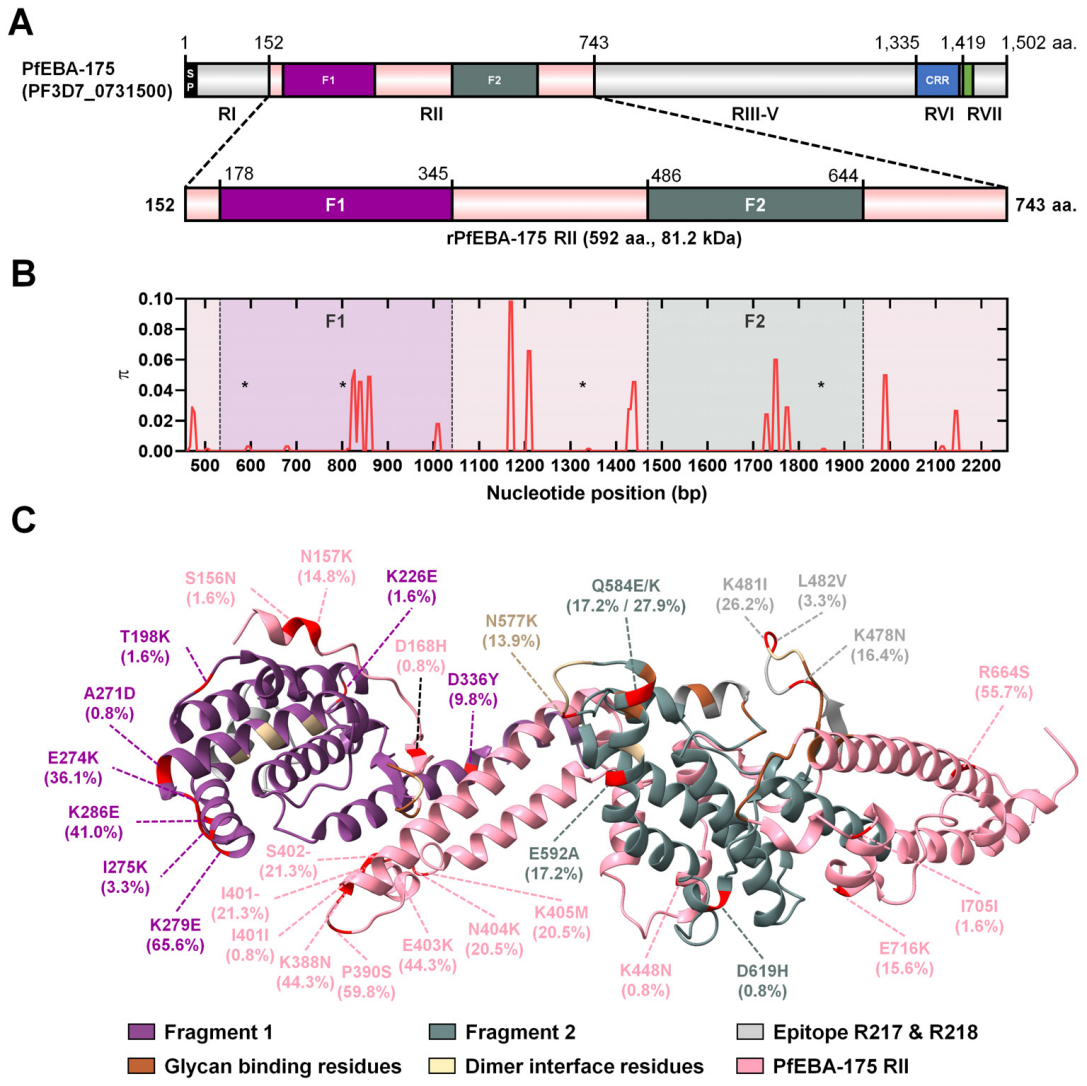
Schematic diagram of PfEBA-175 region II (RII) genetic diversity and protein tertiary structure. **(A)** Schematic representation of the full-length *pfeba-175* gene (PF3D7_0731500), which comprises 4,509 base pairs. Region II (RII) spans 1,776 base pairs and encodes 592 amino acids, with a predicted molecular weight of 81.2 kDa. All regions of PfEBA-175 are depicted, with emphasis on RII. The recombinant protein expression construct (rPfEBA-175 RII) includes residues 152–743. **(B)** Nucleotide diversity (π) across *pfeba-175 RII*, plotted by nucleotide positions, reveals polymorphic sites and distinct diversity peaks. Novel mutation sites at positions 198, 271, 448, and 619, are marked with a star. **(C)** Tertiary structure of PfEBA-175 RII (PDB ID: 1ZRO), showing the two erythrocyte binding-like (EBL) domains: fragment 1 (F1, purple) and fragment 2 (F2, green). Glycan-binding sites are highlighted in brown, dimer interface residues in khaki, and B-cell epitopes targeted by R217 and R218 monoclonal antibodies in grey. All mutations, including non-synonymous, synonymous, and deletions, are marked in red. Mutations within the R217 B-cell epitope region (K478N, K481I, L482V) and dimer interface (N577K) are additionally annotated in grey and khaki, respectively. The glycophorin A (GpA) binding interface remains conserved across clinical isolates and is outlined in bold.

The *pfeba-175* gene (PF3D7_0731500) spans 4,509 base pairs, consists of four exons, and is located on chromosome 7. Region II (RII) comprises 1,776 base pairs encoding 592 amino acids, with a predicted molecular weight of approximately 81.2 kDa (**Figure 2A**). Nucleotide diversity (π) analysis of the RII region revealed that the polymorphic sites were evenly distributed across all domains (**Figure 2B**). The tertiary structure of PfEBA-175 RII was obtained from the Protein Data Bank (PDB ID: 1ZRO), and key structural features were visualized using a color map, highlighting the EBL domains (F1 and F2), glycan-binding sites, dimer interface residues, and B-cell epitopes recognized by the R217 and R218 monoclonal antibodies (32) (**Figure 2C**). Within the RII region, two synonymous mutations were identified at I401I (c.ATT>ATA, 0.8%) and I705I (c.ATA>ATC, 1.6%), and two deletion mutations were observed at I401 (21.3%) and S402 (21.3%) (**Supplementary Table S3**). The positions of deletion mutations were excluded from further analysis of natural selection. In total, 27 non-synonymous mutations were detected in the RII region, with high population frequencies (>50%) for K279E (65.6%), P390S (59.8%), and R664S (55.7%) (**Figure 2C**). Among these non-synonymous mutations, Q584E/K (17.2%/27.9%) represented multiple allelic substitutions. Additionally, four novel non-synonymous mutations, T198K (1.6%), A271D (0.8%), K448N (0.8%), and D619H (0.8%) were identified. Several non-synonymous mutations were located in the F1 domain, including T198K, K226E, A271D, E274K, I275K, K279E, K286E, and D336Y. Additional mutations in the F2 domain included N577K, Q584E, and E592A, D619H (**Figure 2C**; **Supplementary Table S3**). None of the mutations were located within the glycan-binding residues. Importantly, mutations at K478N (16.4%), K481I (26.2%), and L482V (3.3%) were found within the R217 epitope, which is possibly implicated in invasion inhibition. Among them, L482V was mapped to a region overlapping the R217 epitope and the dimer interface. Additionally, N577K (13.9%) was also located at the dimer interface (**Figure 2C**; **Supplementary Table S3**).

### Natural selection in PfEBA-175 RII

Sequence analysis of the *pfeba-175 RII* gene from 122 clinical isolates revealed 26 polymorphic sites and a total of 27 mutations. These included a non-synonymous substitution at position 584 (Q584E/K), where multiple amino acid changes were observed at a single site (**Table 1**). The average of overall nucleotide diversity (*π*) in the study population was revealed 0.00359 ± 0.00012 (*π* ± S.D.). Based on the region, Geita and Kigoma also showed similar nucleotide diversity (*π*) that 0.00354 ± 0.00019 and 0.00365 ± 0.00015, respectively (**Table 1**). Additionally, comparable levels of nucleotide diversity (*π*) were observed at the district level from 0.00348 ± 0.00028 (Nyang’hwale) to 0.00369 ± 0.00026 (Chato). And at the village level from 0.00319 ± 0.00071 (KKB) to 0.00394 ± 0.00032 (GCR) (**Table 1**). At the micro-geographical scale, nucleotide diversity was consistently high and relatively uniform across villages.

**Table 1:** Estimates of nucleotide diversity, haplotype diversity and neutrality indices of PfEBA-175 RII based on the geographical location in Tanzania.

Location	No. of samples	SNPs	No. of haplotype	Diversity ± S.D.		Tajima’s D	Fu and Li’s		Fu’s Fs
				Haplotype (Hd)	Nucleotide (*π*) X 10^-3^		D*	F*	
Geita	**66**	**22**	**32**	**0.931 ± 0.020**	**3.54 ± 0.19**	**0.91836**	**0.32761**	**0.6456**	**-13.496**
Nyang’hwale	**37**	**21**	**23**	**0.940 ± 0.026**	**3.48 ± 0.28**	**0.57243**	**0.12942**	**0.32664**	**-9.26**
GNN	24	20	15	0.924 ± 0.039	3.39 ± 0.38	0.24522	0.23873	0.28146	-3.772
GNK	13	17	11	0.974 ± 0.039	3.64 ± 0.47	0.46486	0.55844	0.60951	-3.525
Chato	**29**	**18**	**15**	**0.926 ± 0.029**	**3.69 ± 0.26**	**1.22135**	**1.28909**	**1.48864**	**-2.165**
GCR	14	17	9	0.934 ± 0.045	3.94 ± 0.32	0.96515	0.78144	0.95242	-0.44
GCI	15	17	11	0.943 ± 0.045	3.52 ± 0.42	0.50815	0.76148	0.79529	-2.477
Kigoma	**56**	**23**	**31**	**0.942 ± 0.021**	**3.65 ± 0.15**	**0.75371**	**0.10634**	**0.40059**	**-14.105**
Kibondo	**25**	**19**	**14**	**0.867 ± 0.061**	**3.54 ± 0.25**	**0.65742**	**0.44198**	**0.59484**	**-2.331**
KKK	15	17	9	0.905 ± 0.054	3.66 ± 0.26	0.68979	0.5017	0.63623	-0.342
KKB	10	17	6	0.778 ± 0.137	3.19 ± 0.71	-0.52704	-0.64011	-0.68975	0.676
Kasulu	**31**	**21**	**22**	**0.974 ± 0.015**	**3.68 ± 0.21**	**0.63533**	**0.78466**	**0.86691**	**-9.744**
KKN	17	19	12	0.949 ± 0.037	3.93 ± 0.23	0.69572	0.81936	0.90644	-2.331
KKM	14	16	13	0.989 ± 0.031	3.41 ± 0.31	0.53882	0.73204	0.77905	-6.644
Overall	**122**	**26**	**51**	**0.935 ± 0.015**	**3.59 ± 0.12**	**0.78498**	**0.40105**	**0.66211**	**-30.614**

The bold text indicates the region and district level.

Neutrality tests were conducted to assess whether natural selection has shaped the polymorphism observed in the *pfeba-175 RII* gene. Intra-species analysis showed weakly positive values for Tajima’s D (0.78498), Fu and Li’s D* (0.40105), and Fu and Li’s F* (0.66211), which may suggest balancing selection or population structure within the species (**Table 1**). However, none of these values were statistically significant, indicating no strong deviation from neutrality. In contrast, Fu’s Fs (-30.614) showed a highly negative value, providing strong evidence for recent population expansion (**Table 1**). To further assess selection pressure, a Z-test comparing the rates of non-synonymous and synonymous substitution (d_N_–d_S_ = 3.15, *p* < 0.001) revealed a significant excess of non-synonymous changes, consistent with action of positive selection (**Table 2**). Inter-species analysis using the McDonald–Kreitman (MK) test with *P. reichenowi* as an outgroup revealed significant purifying selection (NI = 11.258, *p* = 0.003125) (**Table 2**). Taken together, these results suggest that *pfeba-175 RII* has likely undergone recent population expansion and may be subject to positive selection acting on non-synonymous sites across multiple alleles within *P. falciparum*. While the gene appears to be under functional constraint between species, it may be evolving under different selective pressures within the species.

**Table 2:** McDonald-Kreitman (MK) test on PfEBA-175 RII with PrEBA-175 RII as out-group species and d_N_-d_S_ ratio.

Gene	Polymorphic changes within *P. falciparum*		Fixed differences between *P. falciparum* and *P. reichenowi*		Neutrality index (*p* value)	d_N_-d_S_ (*p* value)
	Syn	Non-syn	Syn	Non-syn		
EBA-175 RII	1	27	42	97	11.258 (< 0.01)	3.15(< 0.001)

**Figure 3:**
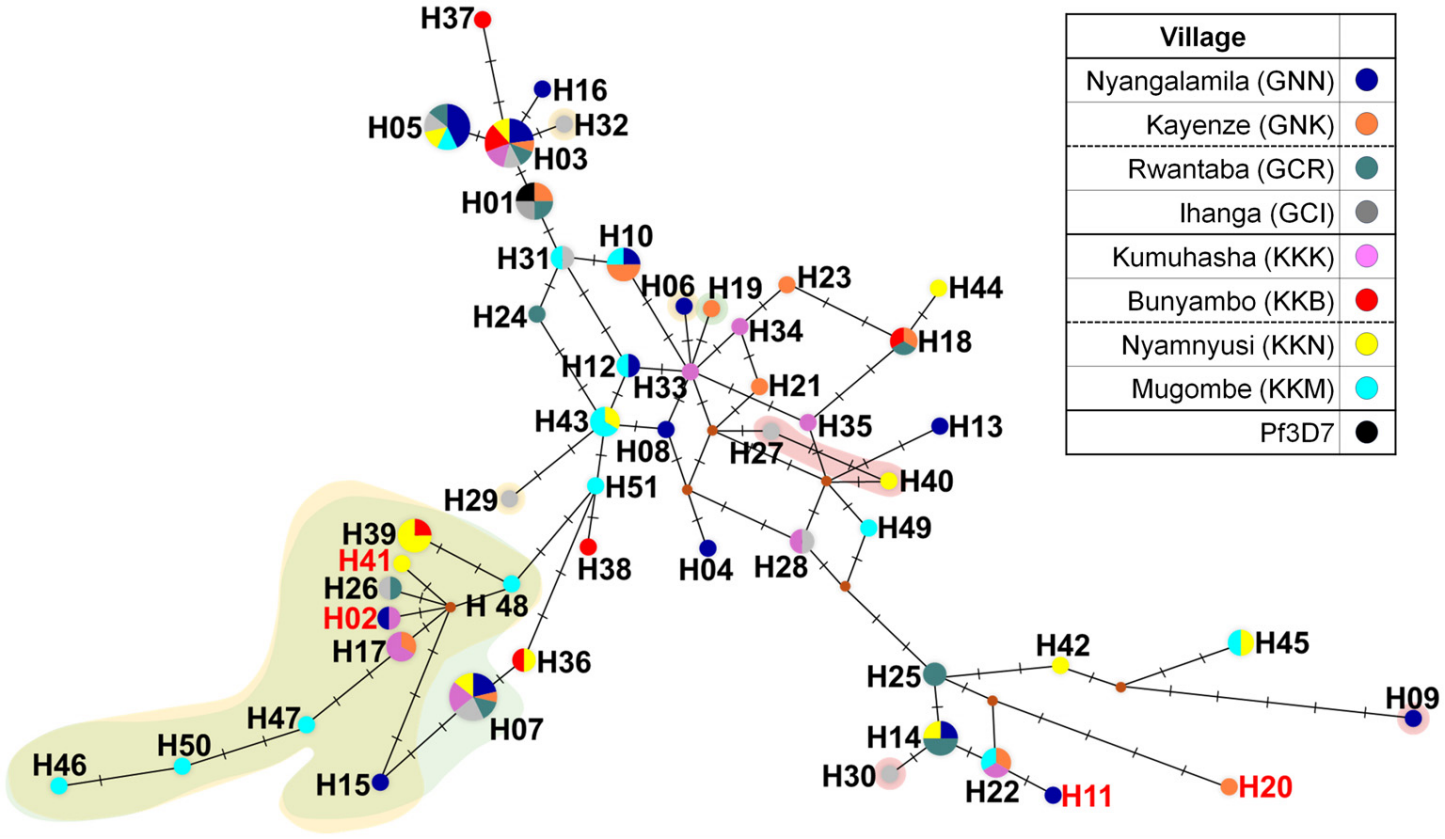
Median-joining haplotype networks of PfEBA-175 RII. The haplotype network was constructed using 122 geographically diverse clinical isolates based on *pfeba-175 RII* sequences using the median-joining algorithm in NETWORK 10.2 software. A total of 51 distinct haplotypes were identified. Node size is proportional to the number of isolates sharing each haplotype, and connecting lines represent mutational steps. Background colors indicate specific substitutions within the R217 monoclonal antibody epitope: yellow (K478N), green (K481I), and red (L482V). Haplotypes H02, H11, H20, and H41 (labeled in red text) contain novel non-synonymous mutations.

A total of 51 distinct haplotypes were identified from the amino acid sequences of 122 clinical isolates, demonstrating substantial genetic diversity within the *pfeba-175 RII* gene (**Table 1** and **Figure 3**). This diversity was largely attributed to a high number of singleton haplotypes (*n* = 32), which supports the scenario of recent population expansion. The remaining 19 haplotypes were shared among multiple isolates and exhibited geographically mixed distributions, suggesting gene flow across different regions. At the village level, Mugombe (KKM) exhibited the highest haplotype diversity, with 14 isolates represented by 13 distinct haplotypes, whereas Bunyambo (KKB) showed the lowest diversity, with six haplotypes (**Figure 3**). The novel mutation T198K (H02), A271D (H41), K448N (H11), and D619H (H20) were identified in specific haplotypes originating from villages, including Nyangalamila (GNN), Kayenze (GNK), Kumuhasha (KKK), and Nyamnyusi (KKN), respectively (**Figure 3**). In addition, three non-synonymous substitutions within the R217 monoclonal antibody epitope such as K478N (16.4%), K481I (26.2%), and L482V (3.3%), observed in isolates from diverse geographic locations. Especially, K478N substitution was detected independently in isolates from H32 (GCI), H06 (GNN), H29 (GCI), and showed a contiguous spread originating from H48 (KKM) (**Figure 3**). The K481I mutation, which frequently co-occurred with K478N, also appeared to have spread contiguously from H48 (KKM). In contrast, the L482V substitution exhibited an independent distribution pattern, occurring in isolates from H09 (GNN), H27 (GCI), H30 (GCI), and H40(KKN) (**Figure 3**).

### Humoral immune response to PfEBA-175 RII

**Figure 4:**
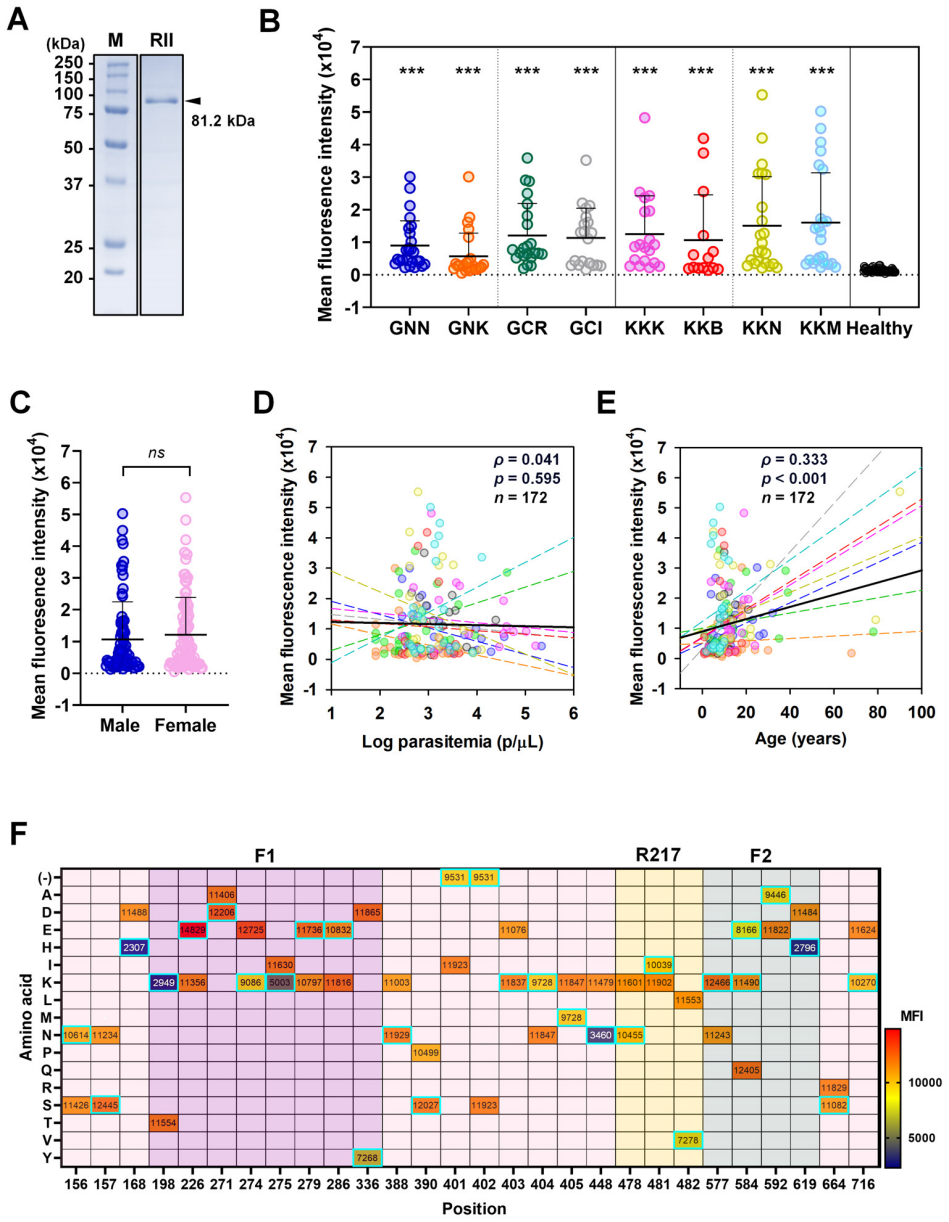
Humoral immune response to PfEBA-175 RII. **(A)** SDS-PAGE analysis confirming purity of recombinant PfEBA-175 RII (592 aa., 81.2 kDa). **(B)** Total IgG levels against PfEBA-175 RII were measured in sera from individuals in the study villages and healthy controls. Mean fluorescence intensity (MFI) ± standard deviation (SD) is shown. Statistical significance was assessed using an unpaired Student’s t-test and significance indicated by triple asterisks (***, p < 0.001). All patient groups exhibited significantly higher IgG responses compared to healthy controls. **(C)** Comparison of IgG levels by gender and were showing no significant correlation (*ns*). **(D, E)** Correlation between MFI with Log parasitemia (parasites/μL) and age (years), respectively. Spearman’s correlation test (*ρ*) was used to assess the association in both cases. Each colored dot represents an individual from a specific village, with dashed lines indicating village-specific trends. The black solid line represents the overall regression line across all villages. **(F)** Impact of amino acid substitutions on antibody binding across PfEBA-175 RII. A heatmap displays the mean MFI values corresponding to amino acid variants at each position. Each cell represents the average MFI for a given residue. The amino acid substitutions from the Pf3D7 reference sequence are highlighted with cyan boxes. Notably, positions 168, 198, 275, 448, and 619 showed substantial changes in MFI upon specific substitutions, suggesting their importance in epitope presentation. In contrast, the introduction of a positive charge at positions 226, 271, 279, 388, 390, 403, and 577 was associated with enhanced antibody binding.

To assess humoral immune responses, recombinant PfEBA-175 RII (81.2 kDa) was expressed in a soluble form and used to screen sera from asymptomatic individuals (**Figure 4A**). Each serum sample was tested in duplicate wells, and fluorescence signals were averaged to obtain the mean fluorescence intensity (MFI). Total IgG reactivity against PfEBA-175 RII was significantly higher in asymptomatic individuals (MFI ± S.D., 11,526 ± 11,707) compared to healthy controls (1,384 ± 573.3) (**Table 3**). Overall assay sensitivity for detecting PfEBA-175 RII–specific IgG in asymptomatic individuals was 84.8% (95% CI, 78.7–89.4), and specificity in healthy controls was 92.1% (95% CI, 82.9–96.6)(**Table 3**). When stratified by region, IgG response was similar between Kigoma (85.0%) and Geita (84.7%) (**Table 3**). However, at the village level, the seropositivity varied substantially. Most villages showed over 90% sero-positivity, whereas Bunyambo (KKB) and Kayenze (GNK) exhibited notably lower IgG responses at 50.0% and 58.3%, respectively (**Figure 4B**; **Table 3**). Analysis of demographic variables revealed no significant differences in IgG responses between gender or parasitemia (*ρ* = 0.041, *p* = 0.595) (**Figures 4C, D**). In contrast, a significant positive correlation was observed with age (*ρ* = 0.333, *p* < 0.001), suggesting that antibody response may be strengthened with repeated exposure over time (**Figure 4E**).

**Table 3:** Humoral immune responses against PfEBA-175 RII-ecto proteins.

Antigen	No. of patient sample			95% CI^b^	MFI^c^	No. of healthy sample			95% CI	MFI	*p* value^e^
	Pos.	Neg.	Total (%)^a^			Pos.	Neg.	Total (%)^d^			
Total	**146**	**26**	**172 (84.8)**	**78.7-89.4**	**11,526**	**5**	**59**	**64 (92.1)**	**82.9-96.6**	**1,384**	**< 0.001**
Geita	**78**	**14**	**92 (84.7)**	**76.0-90.7**	**9,386**						
Nyang’hwale	**38**	**12**	**50 (76.0)**	**62.5-85.7**	**7,410**						
Nyangalamila	24	2	26 (92.3)	75.8-97.8	8,971						
Kayenze	14	10	24 (58.3)	38.8-75.5	5,718						
Chato	**40**	**2**	**42 (95.2)**	**84.2-98.6**	**11,740**						
Rwantaba	21	1	22 (95.4)	78.2-99.1	12,077						
Ihanga	19	1	20 (95.0)	76.3-99.1	11,368						
Kigoma	**68**	**12**	**80 (85.0)**	**75.5-91.2**	**13,987**						
Kibondo	**25**	**8**	**33 (75.7)**	**58.9-87.1**	**11,731**						
Kumuhasha	18	1	19 (94.7)	75.3-99.0	12,515						
Bunyambo	7	7	14 (50.0)	26.8-73.2	10,667						
Kasulu	**43**	**4**	**47 (91.4)**	**80.0-96.6**	**15,571**						
Nyamnyusi	21	2	23 (91.3)	73.2-97.5	15,081						
Mugombe	22	2	24 (91.6)	74.1-97.6	16,040						

a Sensitivity: percentage of positive in patient samples.

b CI, confidence interval.

c MFI, mean fluorescence intensity.

d Specificity, percentage of negative in healthy samples.

e Difference in the total IgG prevalence for each antigen between falciparum patient and healthy individuals were calculated with Mann-Whiteny *U*-test, A *p* value of < 0.05 is considered statistically significant. The bold text indicates the region and district level.

### Impact of amino acid substitutions on antibody binding activity

To evaluate the impact of sequence variation on antibody recognition, mean fluorescence intensity (MFI) values were compared across various amino acid substitutions at multiple positions (**Figure 4F**). Several substitutions resulted in marked reductions in MFI, suggesting potential involvement in antibody binding. Notable examples include D168H, K448N, T198K, and I275K in the F1 domain, as well as D619H in the F2 domain. At 168 and 619, substitution of aspartic acid (MFI = 11,488 and 11,484, respectively) with histidine (2,307 and 2,796, respectively) resulted in dramatic decreases in MFI, indicating the importance of these residues in maintaining epitope integrity (**Figure 4F**). These findings imply that negatively charged residues at these sites may play a structural or electrostatic role critical for antibody binding. Similarly, the substitution of threonine with lysine at position 198 and isoleucine with lysine at position 275 led to a notable reduction in MFI, suggesting that charge alterations at these positions disrupt antibody recognition. In contrast, at positions 226, 271, 279, 388, 390, 403, and 577, the introduction of a positive charge enhanced antibody binding, indicating that in certain contexts, charge addition can be beneficial. This further supports the importance of charge alterations at specific positions for antibody recognition on PfEBA-175 RII (**Figure 4F**).

Many other positions tolerated substitutions without substantial changes in MFI, suggesting limited involvement in direct antibody contact, including the R217 epitope (**Figure 4F**). Collectively, these findings highlight key residues that contribute to the antibody binding interface, either through direct interaction or by stabilizing the structural conformation necessary for effective antibody binding.

## Discussion

Asymptomatic *Plasmodium falciparum* infections are commonly prevalent in high-transmission settings and constitute a major reservoir hence sustaining malaria transmission within communities (21). Although parasitemia is typically low in such infections, prolonged and repeated exposure to blood-stage antigens contributes to the development of naturally acquired immunity (33). This continuous antigenic stimulation may drive parasite genetic diversification and modulate host immune recognition. Despite its importance, most studies on the genetic and antigenic properties of PfEBA-175 have focused on symptomatic infections (17, 34). In contrast, the present study analyzed isolates from asymptomatic individuals to better understand this vaccine candidate in a different epidemiological context. Notably, parasite populations often exhibit substantial genetic variation even within limited geographic distances (25, 35). Such micro-geographic variation is particularly important as it influences the distribution of immune-relevant variants, which can affect the efficacy of vaccines targeting blood-stage antigens (24–26). Therefore, high-resolution analyses of antigen diversity in asymptomatic populations are essential to inform the design of broad protective interventions.

In this study, the genetic diversity and antigenicity of PfEBA-175 RII were analyzed using asymptomatic *P. falciparum* isolates collected from eight Tanzanian villages in areas of high malaria endemicity. A total of 26 amino acid substitutions and 2 deletions were identified in PfEBA-175 RII among these isolates. This number exceeds the 21 substitutions previously reported in isolates from Myanmar, which exhibited the highest diversity in earlier global studies across 12 countries (17). This elevated number of amino acid substitutions may reflect immune pressure resulting from prolonged and repeated antigen exposure typical of asymptomatic infections (36, 37). The range of nucleotide diversity across villages (π ± S.D. = 0.00319 ± 0.00071 to 0.00394 ± 0.00032) is comparable to values reported from other countries, suggesting that immune-driven diversification can occur even at the micro-geographic level in high-endemic settings (17). Reflecting this immune pressure, higher polymorphism was observed in the F1 domain, which is likely exposed to host antibodies (6, 17). In contrast, the relatively lower genetic substitution in the F2 domain suggests functional constraints, likely due to its essential role in GpA binding (6, 38). Although both domains are recognized by monoclonal antibodies, sequence variation appears to be more tolerated in F1 than F2 (32).

Globally common amino acid substitutions such as K279E (65.6%), E403K (44.3%), K481I (26.2%), Q584K (17.2%), and R664S (55.7%) were also frequently observed in isolates from asymptomatic cases in Tanzania, indicating that these residues are subject to widespread immune pressure across different countries (17, 39). Additionally, a deletion of residues I401 (21.3%) and S402 (21.3%) was identified in the Tanzanian samples. Interestingly, both synonymous and deletion mutations were observed at position I401, suggesting that this residue may tolerate genetic and antigenic variability. The presence of a synonymous mutation raises the possibility of functional relevance at the RNA level, while the relatively high frequency of deletion (21.3%) may indicate a potential adaptive advantage or relaxed functional constraint at this site. This deletion, previously reported in isolates from African (Nigeria and Kenya) and Southeast Asian (Myanmar and Thailand) but absent in South American samples, suggests that regional differences in host immunity or parasite population structure may influence its distribution (17). These findings also support the presence of region-specific immune selection, where certain polymorphisms are selectively maintained due to their roles in immune evasion or adaptation to host genetic backgrounds (17). Together with these amino acid substitutions, the natural selection landscape of PfEBA-175 RII in Tanzanian isolates indicates strong immune-driven adaptation. Neutral microsatellite profiling shows similarly high diversity in asymptomatic and symptomatic infections, implying that PfEBA−175 RII selection signatures arise against a broadly diverse population background (40). This is supported by a significantly positive d_N_-d_S_ value (3.15, *p* < 0.001), indicative of positive selection, and a highly negative Fu’s Fs value (-30.614), pointing to recent population expansion or a selective sweep. These findings imply that both adaptive evolution and demographic processes have contributed to the diversification of this antigen, even at the micro-geographic scale, consistent with observations at the national and global levels (17, 34). In line with this, haplotype analysis identified 51 unique haplotypes among the Tanzanian isolates, with 63.0% (*n* = 32) classified as singleton variants, further supporting population expansion through random allele substitution. Notably, this study reports for the first time clinical isolates identical to the 3D7 reference strain, a finding not previously observed in global PfEBA-175 RII populations (17).

PfEBA-175 RII has been shown to induce strong antibody responses in symptomatic infections (6). Similarly, asymptomatic cases in the present study also exhibited high antigenicity, with an overall sensitivity of 84.8%. However, antibody responses varied geographically. Notably, two villages, Bunyambo (KKB, 50.0%) and Kayenze (GNK, 58.3%) exhibited comparatively lower reactivity. Parasitemia in these villages was lower than in other areas, suggesting a potential reduction in antigenicity although our study revealed no statistical significance (41). The overall study population exhibited both high antigenicity and high nucleotide diversity. These immune response patterns resemble those of other surface-localized antigens, such as MSP1 and CSP, which are highly polymorphic but elicit strong immune responses (18, 42, 43). In contrast, several microneme-localized ligand antigens, including PfRh5, PvRBP1a-RII+III, or PvEBP-RII, show high polymorphism often associated with reducing antigenicity, likely due to immune evasion mechanisms (24–26). Interestingly, despite being a microneme-localized ligand, PfEBA-175 RII maintains a high immunogenicity comparable to surface antigens.

Consistent with this, the present study identified several mutations within key B-cell epitopes, including K478N (16.4%), K481I (26.2%), and L482V (3.3%), all located in region targeted by the monoclonal antibody R217 binding (32). Among these, the K478N mutation alters charge and structural properties of the epitope, however, it does not significantly affect antibody recognition. K481I and L482V are considered structurally silent mutations with no predicted impact on the epitope confirmation, although they reduce antibody recognition by 24.9% and 42.9%, respectively (32, 44). Additionally, mutation N577K was found at the dimer interface, which may have functional implications but does not affect naturally acquired antibody recognition (6, 32). Significant mutations affecting antibody recognition were identified at position 157, 168, 275, 448, and 619, all of which involve charged amino acids. alter charged amino acids. These findings suggest that alterations in charge play a key role in naturally acquired antibody recognition. Interestingly, key residues involved in GpA binding are conserved across all study populations (6). These mutations reflect an evolutionary strategy of altering immune-exposed regions while conserving essential function, which poses challenges for vaccine development. Furthermore, age was positively associated with PfEBA-175 RII specific antibody response, likely reflecting cumulative exposure and the gradual acquisition of immunity in high-transmission settings (45). No significant associations were observed with parasitemia or gender, indicating that long-term exposure is a more important factor in shaping PfEBA-175 RII specific immune responses than current infection status. Consistent with this, neutral markers reveal no significant diversity difference by clinical status, aligning with a model where cumulative exposure and multiclonal carriage shape PfEBA−175 RII specific immunity (40).

In conclusion, this study reveals substantial genetic and antigenic diversity of PfEBA-175 RII in asymptomatic *P. falciparum* infections from high-transmission settings in Tanzania. Despite high polymorphism, key regions involved in erythrocyte invasion remained conserved. The high overall antigenicity observed among these isolates suggest that naturally acquired immunity to PfEBA-175 RII is maintained even in the presence of genetic variation. However, regional differences in antibody reactivity, together with the present of mutations involving charged amino acids within known B-cell epitopes, highlight the importance of considering local immune landscapes when evaluating vaccine candidates. These results support the continued investigation of PfEBA-175 RII as a blood-stage vaccine target and emphasize the importance of integrating genetic, immunological, and epidemiological data from asymptomatic infections to inform the design of broadly effective malaria vaccines (21).

## Data Availability

The original contributions presented in the study are included in the article/**Supplementary Materials**. Further inquiries can be directed to the corresponding author/s.
