# Supplementary material for: Micro-geographic variation in antigenic diversity of PfEBA-175 region II in asymptomatic Plasmodium falciparum infections in Tanzania
Source: Front Immunol. 2025 Oct 24;16:1656267. doi: 10.3389/fimmu.2025.1656267 (PMC12592157; doi:10.3389/fimmu.2025.1656267)
Supplement: Supplementary file 1 [file DataSheet1.pdf]

Supplementary file

Micro-geographic variation in antigenic diversity of PfEBA-175 region II in asymptomatic *Plasmodium falciparum* infections in Tanzania

Jadidan Hada Syahada[1], Wang-Jong Lee[1], Hojong Jun[1], Johnsy Mary Louis[1], Fadhila Fitriana[1], Fauzi Muh[2], Feng Lu[3], Md Atique Ahmed[4], Sunghun Na[5], Wanjoo Chun[6], Won Sun Park[7], Bo-Young Jeon[8], Eun-Teak Han[1], Jim Todd[9,10], Alphaxard Manjurano[11], Winifrida Kidima[12], Ernest Mazigo[1,11], Se Jin Lee[5*], and Jin-Hee Han [1,2,13*]

[1] Department of Medical Environmental Biology and Tropical Medicine, School of Medicine, Kangwon National University, Chuncheon, Republic of Korea

[2] Department of Epidemiology and Tropical Diseases, Faculty of Public Health, Universitas Diponegoro, Semarang, Indonesia

[3] Department of Pathogen Biology and Immunology, School of Medicine, Yangzhou University, Yangzhou, China

[4] Malaria Division, Indian Council of Medical Research (ICMR)-Regional Medical Research Centre, Dibrugarh, Assam, India

[5] Department of Obstetrics and Gynecology, Kangwon National University Hospital, Chuncheon, Republic of Korea

[6] Department of Pharmacology, School of Medicine, Kangwon National University, Chuncheon, Republic of Korea

[7] Department of Physiology, School of Medicine, Kangwon National University, Chuncheon, Republic of Korea

[8] Department of Biomedical Laboratory Science, College of Software and Digital Healthcare Convergence, Yonsei University, Wonju, Republic of Korea

[9] Department of Population Health, London School of Hygiene and Tropical Medicine, London, UK

[10] Department of Biostatistics, Catholic University of Health and Allied Sciences (CUHAS), Mwanza, Tanzania

[11] Department of Parasitic Diseases, National Institute for Medical Research, Dar es Salaam, Tanzania

[12] Department of Zoology, College of Natural and Applied Sciences, University of Dar es Salaam, Dar es Salaam, Tanzania

[13] Institute of Medical Sciences, Kangwon National University, Chuncheon, Republic of Korea

*Corresponding author

E-mail: han.han@kangwon.ac.kr

Keyword: *Plasmodium falciparum*, PfEBA-175, Blood-stage malaria vaccine, Genetic diversity, Antigenicity

Supplementary Table S1. Primer sequences used for amplification and sequencing of *pfeba-175* region II.

Gene (position)	5' - Primer sequence – 3'	Product size (bp)
**PCR primer**	Forward: TGTTCCTATTAACGCTGTACGTGTG	1,776 bp
	Reverse: TGCTATTTCAGTGCTTTCCTCAGGAAC	
**Sequencing primer**	Reverse: TCCTTTTTGATATTCTTGGTATTGTTTGGC	-
**PfEBA-175 RII In-fusion cloning primer***	Forward: cttgga***<u>GCGGCCGCA</u>***AATAAGTACGTGCCCATCAACG	1,881 bp
	Reverse: gttctc***<u>TGGCGCGCC</u>***GTCCTTCACTTCGGGGCA	

*, Lowercase letters indicate the pTT5 vector backbone sequence; uppercase italic letters indicate restriction enzyme recognition sites for *NotI* and *AscI*.

Supplementary Table S2. Demographic characteristics of study participants in relation to *Plasmodium falciparum* prevalence.

**Region**	**Geita**				**Kigoma**			
**District**	**Nyang'hwale**		**Chato**		**Kibondo**		**Kasulu**	
**Village**	**GNN**	**GNK**	**GCR**	**GCI**	**KKK**	**KKB**	**KKN**	**KKM**
**Total, *n***	26	24	22	20	19	14	23	24
**Age**								
Mean (S.D.)	11.7 (8.6)	14.4 (13.6)	16.0 (17.7)	10.1 (4.0)	12.9 (5.4)	7.7 (5.8)	17.7 (22.0)	7.3 (2.4)
Median	8.5	10.5	10.5	10.0	12.0	8.5	9.0	8.0
Range	1-32	3-68	1-78	4-21	3-24	1-18	5-90	2-12
**Age group, *n* (%)**								
≤5 1	9	5	5	3	2	5	3	10
6-10 2	6	7	6	13	4	5	11	13
11-15 3	5	1	6	3	8	3	4	1
16-20 4	1	8	1	0	3	1	0	0
21-25 5	2	1	0	1	2	0	2	0
25< 6	3	2	4	0	0	0	3	0
**Gender, *n* (%)**								
Female	19	13	11	12	10	8	11	11
Age, mean (S.D.)	12.6 (8.8)	14.3 (7.5)	17.8 (13.9)	10.0 (4.3)	14.2 (5.1)	9.2 (5.6)	19.6 (24.6)	7.8 (2.0)
Male	7	11	11	8	9	6	12	13
Age, mean (S.D.)	9.1 (8.7)	14.7 (18.9)	14.5 (21.4)	10.4 (3.7)	11.4 (5.8)	5.6 (5.6)	15.8 (20.4)	7.0 (2.9)

Supplementary Table S3. Amino acid substitutions in PfEBA-175 region II, their frequencies in the study population, and associated structural or functional sites.

Mutation	Frequency	Structural/Functional Site
Non-synonymous		
S156N	1.6%	N/A
N157S	14.8%	N/A
D168H	0.8%	N/A
T198K	1.6%	F1 Domain
K226E	1.6%	F1 Domain
A271D	0.8%	F1 Domain
E274K	36.1%	F1 Domain
I275K	3.3%	F1 Domain
K279E	65.6%	F1 Domain
K286E	41.0%	F1 Domain
D336Y	9.8%	F1 Domain
K388N	44.3%	N/A
P390S	59.8%	N/A
E403K	44.3%	N/A
N404K	20.5%	N/A
K405M	20.5%	N/A
K448N	0.8%	N/A
K478N	16.4%	Epitope R217 [32]
K481I	26.2%	Epitope R217 [32]
L482V	3.3%	Epitope R217, Dimer interface (F2 β-finger) [32]
N577K	13.9%	F2 Domain, Dimer interface (β-sheet) [6, 32]
Q584E/K	17.2% / 27.9%	F2 Domain
E592A	17.2%	F2 Domain
D619H	0.8%	F2 Domain
R664S	55.7	N/A
E716K	15.6%	N/A
Synonymous		
I401I	0.8%	N/A (c.ATT>ATA)
I705I	1.6%	N/A (c.ATA>ATC)
Deletions		
I401-	21.3%	N/A
S402-	21.3%	N/A
